# Impact of gender on the willingness to participate in clinical trials and undergo related procedures in individuals from an Alzheimer’s prevention research cohort

**DOI:** 10.1186/s13195-024-01626-1

**Published:** 2024-12-19

**Authors:** Lidia Canals-Gispert, Alba Cañas-Martínez, Gema Huesa, Marc Suárez-Calvet Alomà, Marta Milà-Alomà, Eider Arenaza-Urquijo, Davide Cirillo, Annemarie Schumacher Dimech, Maria Florencia Iulita, Julie Novakova Martinkova, Maria Carmela Tartaglia, Frances-Catherine Quevenco, Antonella Santuccione Chadha, Gonzalo Sánchez-Benavides, Carolina Minguillón, Maria Teresa Ferretti, Karine Fauria, Anna Brugulat-Serrat, for the ALFA Study, Jordi Freixa, Juan Domingo Gispert, Oriol Grau-Rivera, Xavier Gotsens, Xavier Meléndez, Tania Menchón, José Luis Molinuevo, Pau Sánchez, Montserrat Vilà

**Affiliations:** 1 Barcelonaβeta Brain Research Center (BBRC), Pasqual Maragall Foundation, Barcelona, Spain; 2 IMIM (Hospital del Mar Medical Research Institute), Barcelona, Spain; 3 Centro de Investigación Biomédica en Red de Fragilidad y Envejecimiento Saludable (CIBERFES), Madrid, Spain; 4 Neurology Department, Hospital del Mar, Barcelona, Spain; 5 Barcelona Supercomputing Center, Life Sciences Department and Bioinfo4Women, Barcelona, Spain; 6 Women’s Brain Project & Foundation, Eptingerstrasee 14, Basel, Switzerland; 7 Department of Health Sciences and Medicine, University of Lucerne, Lucerne, Switzerland; 8 Altoida Inc. Washington DC, Washington DC, USA; 9 Department of Neurology, Second Faculty of Medicine, Memory Clinic, Charles University and Motol University Hospital, Prague, Czech Republic; 10 Tanz Centre for Research in Neurodegenerative Diseases, University of Toronto, Toronto, ON Canada; 11 Krembil Brain Institute, Memory Clinic, University Health Network, Toronto, ON Canada; 12 Global Brain Health Institute, San Francisco, CA USA; 13 Center of Alzheimer’s Research, Karolinska Institutet, Gävlegatan 16, 8th Floor, 113 30 Stockholm, Sweden

**Keywords:** Gender, Clinical trials, Alzheimer’s disease, Precision medicine, Lumbar puncture

## Abstract

**Background:**

Although there is growing evidence of the association between gender and early diagnosis of preclinical Alzheimer's disease, little attention has been given to the enrolment ratio of men and women in clinical trials and data reporting.

**Methods:**

This study aims to analyze gender differences in sociodemographic factors associated with the willingness to participate in clinical trials and undergo specific procedures in the context of an Alzheimer's disease prevention research cohort. 2544 cognitively unimpaired participants from the ALFA parent cohort (age 45–75 years) of the Barcelonaβeta Brain Research Center were contacted through a structured phone call to determine their willingness to participate in Alzheimer's disease clinical trials and undergo trial-related procedures (magnetic resonance imaging, lumbar puncture, positron emission tomography, and cognitive assessment). Sociodemographic data on education, occupational attainment, civil and caregiver status were gathered. Stepwise logistic regression models were performed in order to study the interaction between gender and sociodemographic factors in the willingness to participate in clinical trials and to undergo clinical trial-related procedures.

**Results:**

1,606 out of the 2,544 participants were women (63.1%). Women were significantly younger and had lower educational attainment compared with men. In addition, women were more likely to be caregivers, single and unemployed. Women showed a significantly lower willingness than men to participate in a clinical trial (*p* = 0.003) and to undergo a lumbar puncture (*p* < 0.001). Single women were less willing to participate in clinical trials than single men (*p* = 0.041). Regarding clinical trial-related procedures, women with higher years of education were significantly less willing to undergo a lumbar puncture (*p* = 0.031).

**Conclusion:**

We found gender differences regarding the sociodemographic factors that predict the willingness to participate in clinical trials and to undergo clinical trial-related procedures. Our results highlight the urgent need to design recruitment strategies accounting for gender-related factors, particularly those related to marital status and education.

## Introduction

Alzheimer's disease (AD) represents 60% to 80% of all dementia cases [1]. Due to the progressive aging of the population, this figure is expected to increase to over 150 million by 2050, thus highlighting AD as a global public health priority [2]. AD disproportionately affects women in prevalence and severity [3, 4], and both individuals' sex (biological variable) and gender (socio-cultural variable) have been shown to account for an important part of this AD prevalence variability [5–7].

Sex-related differences have been observed in disease symptoms and progression [8, 9], in biomarkers [5, 10], and in genetic risk [11–14]. The sociodemographic factors have also shown a gender-specific effect on the AD heterogeneity [5, 13, 15]. Women in certain countries and cultures have faced educational inequality resulting in gender disparities in education attainment [16, 17]. A higher risk of developing AD in women has been associated with fewer opportunities for them to attain higher occupational positions compared to men [18]. Another risk factor more prevalent in women is being a primary informal caregiver, shouldering a more significant burden of care [5, 19]. However, despite evidence of sex and gender differences in the epidemiology, pathogenesis, progression, and treatment response of AD, little attention has been given to the gender equality of clinical trials enrolment and the integration of these differences in analyses and study outcomes [20].

Historically, women have been underrepresented in clinical trials across different medical fields. However, this tendency is slowly changing partially due to changes in laws, regulations, policies, and guidelines [21–25]. Recently, R. K. Rechlin et al*.* (2022) surveyed neuroscience and psychiatry papers published in 2009 and 2019 and found that, despite a 30% increase in the percentage of studies including both sexes, in 2019 only 19% of them used an optimal design for the discovery of possible sex differences and only 5% included sex as a discovery variable [26]. Regarding clinical trials enrolment, a cross-sectional study analyzed the enrolment of more than 5 million participants throughout different fields between 2000 and 2020 and found that clinical trials in the field of neurology had the second lowest female participant representation [24]. Similar trends in female underrepresentation were noted in randomized controlled trial of specific neurologic conditions, such as stroke [27].

When looking specifically at AD clinical trials, a recent systematic review and meta-analysis of 56 randomized AD clinical trials with more than 39,000 total participants, 59.0% of participants overall, and a mean of 57.9% in trials of experimental drugs, were women [20]. However, despite the higher proportion of women enrolling in the AD clinical trials analyzed, the percentages did not represent the prevalence of AD among women (about 64% accordingly to Global Burden of Disease 2019 [28]). Pinho-Gomes et al*.* (2022), conducted a study on women representation in dementia clinical trials, revealing that while the proportion of men and women is equal in trials conducted over the past decade, the percentage of women participating is lower than that of women with dementia in the general population [29]. Altogether, these analyses highlight a sex bias persistence and the need to develop strategies to improve diversity in clinical trials enrolment. AD clinical trials face various challenges in recruiting participants, which might create a pre-selection gender bias because of demographic characteristics [30, 31]. In a study with more than 20,000 mild cognitive impairment (MCI) and dementia patients, the authors found that women were less likely to participate in a clinical trial due to older age, less years of education and a higher number of comorbidities when compared to men [31]. Recently, Rosende-Roca et al*.* (2021) designed a study aiming to identify the role of sex in eligibility for participation in dementia trials. They showed that, after applying a set of pre-screening requirements, women had a lower chance of being eligible for enrolment in AD clinical trials than men, and education appeared as the leading cause [19]. Therefore, it is critical to work towards providing equal opportunities and improved access for clinical trial candidates, regardless of gender.

The impact of the interaction between gender and sociodemographic variables on the willingness to undergo diagnostic procedures, particularly in the early stages of the AD *continuum*, is still unclear. In a recent study, Erickson, et al. (2022) found that the willingness of middle-aged and older adult AD research participants to enroll in AD biomarker studies was driven by the biomarkers collection method (being lowest for the cerebrospinal fluid (CSF) biomarkers), as well as research attitudes, and disclosure of individual results [32]. By analyzing barriers, motivations, and facilitators separately for women and men regarding their participation in AD clinical trials, inclusion strategies might be improved, particularly in the early stages of the AD *continuum*. This would also help personalize the AD patients’ pathway, benefiting both clinical practice and clinical trials .

In order to address this gap, we aimed to investigate whether the interaction between gender and sociodemographic variables (such as education, marital status, occupation attainment status, and caregiving) predict the willingness to consider participating in a clinical trial and undergoing clinical trial-related procedures in 2,544 cognitively unimpaired (CU) individuals from the ALFA parent cohort [33], in the context of AD prevention research.

## Material and methods

### Participants and study design

Participants in this study were part of the ALFA (for ALzheimer and FAmilies) parent cohort, a longitudinal monocentric research platform established at the Barcelonaβeta Brain Research Center (BBRC), Barcelona, Spain, to identify pathophysiological alterations in preclinical AD. The ALFA parent cohort includes 2,743 CU middle-aged (45–75 years old) individuals, 47% of them being first-order descendants of AD patients [33]. In brief, participants had a Clinical Dementia Rate score [34] equal to 0 and scored within the established normal cut-offs for the neuropsychological battery that included the Mini-Mental State Examination ≥ 26, Memory Impairment Screen ≥ 6 [34, 35], Time-Orientation subtest of the Barcelona Test II ≥ 68 [36], and semantic fluency (animals) ≥ 12 [37, 38]. Exclusion criteria included major psychiatric disorders, neurological disorders, brain injury that could affect cognition, or family history of AD with a suspected autosomal dominant pattern.

The baseline assessment of the ALFA parent cohort was carried out during 2013–2014. This visit included cognitive and clinical evaluation, lifestyle habits questionnaires, and DNA extraction. In 2016, after the baseline visit (mean of 1.9 years), 2,544 ALFA participants were contacted by a phone call. This call aimed to (1) confirm the participant's interest in being part of the BBRC research platform, (2) register their willingness to consider the possibility of participating in a clinical trial, and (3) register their willingness to undergo clinical trial-related procedures.

### Phone call structure

BBRC research assistants (four women and two men) contacted the participants by phone after the baseline visit, following a script (Supplemental Material, Figure s1). After confirming their interest in BBRC studies and the inclusion criteria for ALFA parent cohort, research assistants checked the willingness to undergo each clinical trial-related procedure (Magnetic Resonance Imaging [MRI], Lumbar Puncture [LP], and Positron Emission Tomography [PET]) with the question: *"Would you accept to undergo/realize an MRI/LP/PET/cognitive assessment, in case the investigation requires it?".* In cases where a participant required it, research assistants briefly explained each procedure. In the cases of an affirmative answer, research assistants checked the contraindications for the specific procedure (e.g., a pacemaker, metal or electronic implant, or carrier of an infusion pump). Then, the research assistant assessed the possibility of considering participating in a future clinical trial with the question: "*BBRC will carry out clinical trials aimed at people who, like you, do not have any cognitive impairment. Do you want us to inform you if we start a clinical trial to consider the possibility of participating?".* Then, in the cases that a participant required it, research assistants gave a more extended explanation of what a clinical trial is (Supplemental Material, Table s1).

### Variables

#### Sociodemographical factors

Sociodemographic factors were gathered during the ALFA parent cohort baseline visit. These included education (years), marital status (groups: single [including separated and widower] and committed relationship [including married, common-law, and stable partner]), occupational attainment (active, temporarily unemployed, unemployed), and caregiver status (defined as a person who currently provides unpaid daily care to a family member with an illness [yes/no]).

#### Statistical analysis

**Fig. 1:**
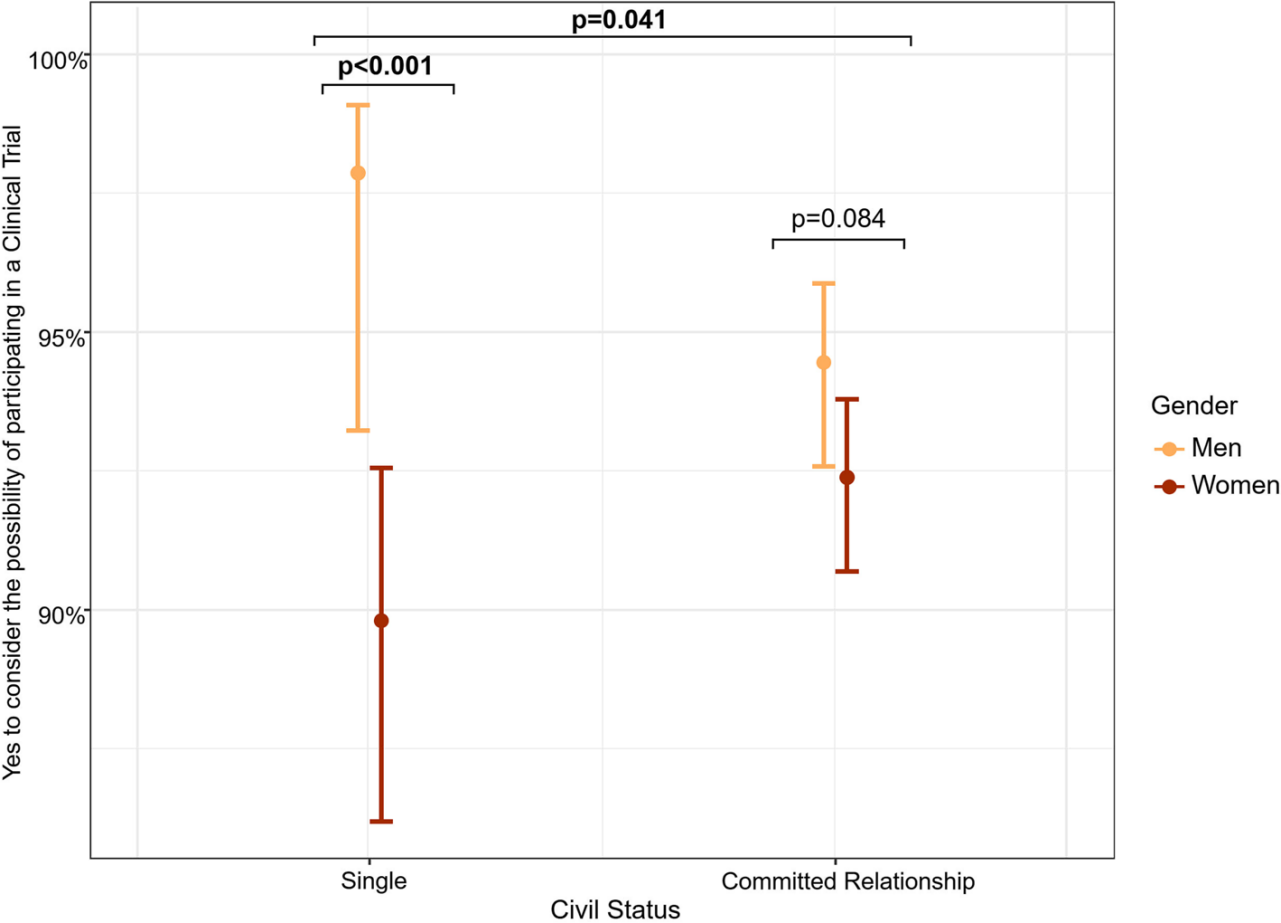
Gender interaction with marital status to predict the willingness to consider participating in a clinical trial. The graph represents the percentage of probability to say yes to consider the possibility of participating in a clinical trial according to marital status by gender. The p-values were assessed by a logistic regression adjusted by age and followed by Tukey corrected pair-wise post hoc comparisons Single women were significantly less willing to consider the possibility of participating than single men (*p* < 0.001)

First, a descriptive analysis of the study sample was performed, dividing the sample by gender. Chi-square test (for categorical variables) or Mann–Whitney U test (for quantitative variables) were used to assess gender differences for each variable. Normality of quantitative variables were assessed by Kolmogorov-Smirnoff tests. Initial analyses to assess gender differences in the percentage of willingness to participate in a clinical trial and each clinical trial-related procedure were performed using chi-square tests. For those procedures that showed significant differences, further backward stepwise logistic regression models were conducted to predict the willingness to participate, with the willingness (yes/no) as the primary outcome, education years, civil, employment and caregiver status the predictors, and, finally, gender and age as the covariates. Initially, all predictors were introduced into the models along with all two-way interactions with gender. Progressively, factors showing *p* > 0.1 were removed from the model. SPSS® 25.0 for Windows was used for all the analyses. Differences were considered to be significant at *p* < 0.05 (Fig 1).

## Results

### Participant's characteristics

From the 2,544 individuals that were contacted by phone, 938 were men (36.9%), with a mean age of 59.63 ± 6.74 years, and 1,606 were women (63.1%), with a mean age of 59.02 ± 6.74 years. The main characteristics of the participants are shown in Table 1. There were significant differences in age (women were younger), level of education (women had lower years of education), occupational attainment status (a higher number of men were active), marital status (the number of singles was higher in women, while the number of committed relationship was higher in men), as well as in caregiving (higher number of current women caregivers).

**Table 1:** Participants’ characteristics by gender

	**Total**	**Men**	**Women**	***p***
**N**	2544	938 (63.9)	1606 (63.1)	
**Age (years)**	59.3 (6.7)	59.63 (± 6.74)	59.02 (± 6.74)	0.026*
**Education (years)**	13.4 (3.51)	13.78 (± 3.47)	13.19 (± 3.52)	< 0.001*
**Marital status**				< 0.001*
Single	518 (20.4)	133 (14.2%)	385 (24.0%)	0.008*
Committed relationship	2025 (79.6)	805 (85.8%)	1220 (76.0%)	< 0.001*
**Occupational attainment**				0.032*
Active	1692 (66.5)	654 (70.3%)	1038 (65.8%)	0.027*
Temporarily unemployed	265 (10.4)	82 (8.8%)	183 (11.6%)	0.248
Unemployed	551 (21.7)	194 (20.9%)	357 (22.6%)	0.323
**Caregiver (yes)**	477 (18.8)	106 (11.3%)	371 (23.1%)	< 0.001*

Notes: Data are expressed a mean (M) and standard deviation (SD) or percentage (%), as appropriate

Regarding the global willingness to participate in clinical trials and to undergo related procedures, we found that 86.0% of participants were willing to consider participating in a clinical trial, 95.4% to undergo an MRI, 81.3% a LP, 91.1% a PET, and 91.9% to undertake cognitive assessment. Regarding gender differences, we found that men were significantly more willing to participate in a clinical trial (X^2^ = 8.965; *p* = 0.003) and to undergo a LP (X^2^ = 11.204; *p* < 0.001) (Table 2). No significant between-group differences were found for other clinical trial-related procedures.

**Table 2:** Distribution of willingness to participate in clinical trials and related procedures

	**Total**	**Men**	**Women**	***p***
Willingness (yes), *n* (%)				
Clinical Trial	2187 (86.0)	832 (88.7)	1355 (84.4)	0.003*
MRI	2426 (95.4)	896 (95.5)	1530 (95.3)	0.729
LP	2068 (81.3)	792 (84.4)	1276 (79.5)	< 0.001*
PET	2318 (91.1)	863 (92.0)	1455 (90.6)	0.202
Cognitive assessment	2338 (91.9)	872 (93.0)	1466 (91.93)	0.182

*Abbreviations*: *MRI* Magnetic Resonance Imaging, *PET* Positron Emission Tomography

^*^p < 0.05

### Interaction between gender and sociodemographic factors on the willingness to participate in a clinical trial

Table 3 shows the logistic regression model results for the interaction between gender and sociodemographic factors on the willingness to consider participating in a clinical trial. The results show that the interaction between gender and marital status significantly predicted the willingness to participate in clinical trials (OR 4.75; 95% CI [1.07–21.18]; *p* = 0.041). Single women were significantly less willing to consider the possibility of participating than men counterparts (*p* < 0.001) (Fig. 1).

**Table 3:** Logistic regression model for the interaction between gender and sociodemographic factors on the willingness to consider participating in a clinical trial

	**B**	**Odds ratio (95% CI)**	***p***
Gender (reference: men)	−3.66	0.03 (0.01 – 0.86)	0.041*
Age	0.01	1.00 (0.97 – 1.03)	0.841
Marital status	−0.44	0.64 (0.30 – 1.37)	0.251
Occupational attainment	0.02	1.01 (0.78 – 1.33)	0.911
Caregiver (yes)	−0.21	0.81 (0.49 – 1.32)	0.394
Education (years)	−0.33	0.97 (0.92 – 1.02)	0.256
Gender*Marital status	1.61	5.02 (1.11 – 22.78)	0.036*
Gender * Occupational attainment	0.07	1.07 (0.67 – 1.71)	0.781
Gender*Caregiver	0.93	2.54 (0.95 – 6.81)	0.063
Gender*Education (years)	0.02	1.02 (0.91 – 1.14)	0.757

^*^p < 0.05

### Interaction between gender and sociodemographic factors on the willingness to undergo a lumbar puncture

Women with higher years of education were significantly less willing to undergo a LP (OR 0.92; 95% CI [0.86–0.99]; *p* = 0.031; Table 4) than men. We found no significant interaction between gender and any other sociodemographic factor.

**Table 4:** Logistic regression model for the interaction between gender and sociodemographic factors on the willingness to undergo a lumbar puncture

	**B**	**Odds ratio (95% CI)**	***p***
Gender (reference: men)	0.64	1.89 (0.71 – 5.03)	0.201
Age	−0.03	0.96 (0.96 – 0.99)	0.003*
Marital status	0.13	1.14 (0.81 – 1.61)	0.440
Occupational attainment	−0.10	0.90 (0.75 – 1.08)	0.254
Caregiver (yes)	−0.16	0.85 (0.60 – 1.21)	0.440
Education (years)	−0.01	0.99 (0.96 – 1.03)	0.516
Gender*Marital status	0.28	1.32 (0.67 – 2.61)	0.420
Gender * Occupational attainment	−0.10	0.99 (0.69 – 1.42)	0.957
Gender*Caregiver	−0.09	0.91 (0.45 – 1.83)	0.794
Gender*Education (years)	−0.79	0.92 (0.86–0.99)	0.031*

*p < 0.05

## Discussion

This is the first study to investigate whether the interaction between gender and sociodemographic factors predicted the willingness to participate in AD clinical trial and undergo clinical trial-related procedures, in the context of an AD prevention cohort. The results showed that women were less willing to consider being involved in AD-related clinical trials and to undergo a LP. Specifically, we observed in women that 1) having a committed relationship predicted a higher willingness to participate in AD clinical trials, and 2) higher levels of education predicted less willingness to undergo a LP.

According to multiple studies, there appears to be a willingness among the general public to take part in research studies, including clinical trials [39–41]. However, this willingness is influenced by a variety of factors, including individual health status, demographics, attitudes towards research, potential personal benefits, and altruistic reasons. Therefore, while there is a willingness to participate, it is not universal and may vary depending on individual circumstances, attitudes, and prior experiences [39–41]. Here, we have focus on the impact of gender on the willingness to participate in clinical trials and undergo related procedures.

Overall, women were less willing to consider participating in a clinical trial than men, particularly women living independently. This result may reflect the shift in the trend in women’s educational access and occupational attainment that has been observed in recent decades. These new opportunities are associated with changes in the responsibility of balancing work and family, historically attributed to women [19]. Therefore, this new situation can pose a challenge, particularly for single women who lack support from a committed partner to balance their personal and family needs. Thus, not being in a committed relationship may influence women’s lower level of willingness and engagement in research, as well as compliance with protocol visits (e.g., the availability of a study partner, which is usually required in AD clinical trials). These factors should also be considered regarding the higher risk of attrition showed in women [42].

A primary diagnostic procedure used in most clinical trials focused on the early stages of AD is the LP, which involves obtaining and sampling CSF from the spinal canal. Despite being a safe clinical procedure, with some minor complications associated such as post-epidural-puncture headaches, LP is generally considered invasive with a negative perception by the population [43]. Thus, the requirement of a LP is a significant barrier to AD research recruitment [44]. Interestingly, we have observed that overall women showed less willingness to undergo this procedure. This finding may be related to a previous negative experience (such as during labor), either personal or reported by a female acquaintance with epidural anesthesia (a procedure with some similarities to LP). In our sample, only 63 women self-reported a previous epidural experience, of whom three declined to undergo LP. However, the longitudinal follow-up of those participants will allow us to assess the impact of this prior experience.

Regarding the interactions between gender and sociodemographic factors, highly educated women showed less willingness to undergo a LP. These findings contradict previous studies that found higher education to be associated with greater willingness to undergo this procedure, however these studies did not examine gender differences in this association [45, 46]. One possible explanation is that a higher level of education may lead to increased awareness of the potential complications associated with the LP procedure. In fact, rates of LP complications are increased in the female population (including both post-epidural-puncture headache [47, 48] as well as potential for more serious complications, such as medullar conus injury) [49]. Nevertheless, serious LP complications are very rare (< 0.01%) [49], and post-epidural-puncture headache risk can be lowered by using appropriate precautions [50]. One way to overcome this barrier could be educational programs to increase willingness to participate in research involving LP [44]. Interestingly, fear of LP has been identified as an independent predictor of LP complications, highlighting the importance of proper education before and during the procedure [51]. Nevertheless, with the advances of blood-based biomarkers as a non-invasive alternative to LP, gender enrolment differences may decrease significantly in the near future.

This study is not free of limitations. Some variables implicated in the analysis, such as education, civil, employment, and caregiving status, were collected during the baseline visit and were not updated afterwards during phone call interviews (2–3 years gap). An additional limitation pertains to the fact that participants were not directly queried about their desired to enroll in a specific ongoing clinical trial. Instead, they were presented with a hypothetical scenario and asked to disclose their intentions if they were to receive an invitation to participate. It should also be noted that, when this information was collected, participants were unaware of potential preclinical conditions such as genetic risk. As such, their responses may be subject to change during subsequent follow-up visits. Other limitation of our study is the ALFA parent cohort characteristics, such the highest enrichment of family history of sporadic AD. However, it has not been considered a consistent predictor for enrolment willingness [52]. Lastly, the possibility of social desirability bias cannot be ruled out, particularly about responses to non-binding hypothetical scenarios, which may affect men and women differently. Nevertheless, extrapolation to the general population should not be done without further research.

Overall, our results support the importance of developing recruitment strategies that consider the interaction between sociodemographic factors and gender, including marital status and education. These findings provide a foundation for future investigations in this area and underscore the potential benefits of incorporating this knowledge into medical research, practice, and participant education to promote gender equity across all domains of health [53]. Additionally, future research should explore a more diverse population and a broader range of sociodemographic factors, as well as conduct a detailed examination of the motivators, barriers, and facilitators of enrolment, in order to gain a deeper understanding of the gender-specific aspects of clinical trial recruitment.

## Conclusion

In the context of an AD prevention cohort, this study provides evidence of gender-based disparities in the sociodemographic factors that predict willingness to consider participation in clinical trials and undergo a LP. Our findings highlight the importance of developing recruitment and enrolment strategies for AD clinical trials that consider gender-related factors, particularly those associated with education and marital status. The ultimate goal is to achieve greater diversity in AD clinical trial enrolment and promote gender equity by ensuring fair representation of the AD population.

## Data Availability

No datasets were generated or analysed during the current study.

## Abbreviations

AD: Alzheimer’s disease
BBRC: Barcelonaβeta Brain Research Center
CI: Confidence interval
CSF: Cerebrospinal fluid
CU: Cognitively unimpaired
ERC: European Research Council
ESF: European Social Fund
LP: Lumbar puncture
M: Mean
MCI: Mild cognitive impairment
MRI: Magnetic resonance imaging
OR: Odds ratio
PET: Positron emission tomography
RCTs: Randomized controlled clinical trials
SD: Standard deviation
